# Three Versions of the Perceived Stress Scale: Psychometric Evaluation in a Nationally Representative Sample of Chinese Adults during the COVID-19 Pandemic

**DOI:** 10.3390/ijerph18168312

**Published:** 2021-08-05

**Authors:** Zhuang She, Dan Li, Wei Zhang, Ningning Zhou, Juzhe Xi, Kang Ju

**Affiliations:** 1 Shanghai Key Laboratory of Mental Health and Psychological Crisis Intervention, Affiliated Mental Health Center (ECNU), School of Psychology and Cognitive Science, East China Normal University, Shanghai 200062, China; cdsz_2008@yeah.net (Z.S.); danli2021@yeah.net (D.L.); nnzhou@psy.ecnu.edu.cn (N.Z.); jukang2009@163.com (K.J.); 2 Shanghai Changning Mental Health Center, Shanghai 200335, China; 3 Mental Health Center, Hainan Medical University, Haikou 571199, China; 4 Mental Health Center, Wuhan Polytechnic University, Wuhan 430074, China; iris20190009@126.com

**Keywords:** Chinese Perceived Stress Scale, reliability, validity, general population, COVID-19

## Abstract

(1) Background: The COVID-19 outbreak has created pressure in people’s daily lives, further threatening public health. Thus, it is important to assess people’s perception of stress during COVID-19 for both research and practical purposes. The Perceived Stress Scale (PSS) is one of the most widely used instruments to measure perceived stress; however, previous validation studies focused on specific populations, possibly limiting the generalization of results. (2) Methods: This study tested the psychometric properties of three versions of the Chinese Perceived Stress Scale (CPSS-14, CPSS-10, and CPSS-4) in the Chinese general population during the COVID-19 pandemic. A commercial online survey was employed to construct a nationally representative sample of 1133 adults in Mainland China (548 males and 585 females) during a one-week period. (3) Results: The two-factor (positivity and negativity) solution for the three versions of the CPSS showed a good fit with the data. The CPSS-14 and CPSS-10 had very good reliability and the CPSS-4 showed acceptable reliability, supporting the concurrent validity of the CPSS. (4) Conclusions: All three versions of the CPSS appear to be appropriate for use in research with samples of adults in the Chinese general population under the COVID-19 crisis. The CPSS-10 and CPSS-14 both have strong psychometric properties, but the CPSS-10 would have more utility because it is shorter than the CPSS-14. However, the CPSS-4 is an acceptable alternative when administration time is limited.

## 1. Introduction

People may feel stressed when they perceive a situation to be threatening but lack effective resources to cope with the threat [1]. This explanation fits with the high stress reported by Chinese in the context of the current COVID-19 crisis [2]. A similar situation was also found in previous pandemics. For example, the general public in Hong Kong demonstrated increased stress in their family and work settings under the severe acute respiratory syndrome (SARS) crisis [3]. Pandemics bring unprecedented social fear about a virus that is highly infectious and potentially deadly, together with quarantine, potential economic losses, and conflicting and inadequate information that the public obtain on the internet [2,4,5]. All of these may make people experience stress. Stress during the COVID-19 crisis may further decrease people’s well-being and increase other health-related problems such as anxiety and depression [6,7]. The current crisis in China allows us to test the psychometric properties of a measure of perceived stress in a high stress context.

The Perceived Stress Scale (PSS) is one of the most widely used stress perception assessment instruments in the world [8]. The scale was originally developed in 1983 by Cohen et al. [9] and was designed to assess the degree of stress people felt in unpredictable, out-of-control, and overloaded situations. The original version of the PPS had 14 items (PSS-14) with seven negative items (e.g., “Unable to control the important things in your life?”) and seven positive items (e.g., “Confident about your ability to handle your personal problems?”). Based on factor analysis, the researchers removed the four items with the lowest factor loadings on the PSS-14 to create a shortened 10-item version (PPS-10) [10]. An even briefer four-item version (PSS-4) was developed for ease of use when there are time constraints on data collection (e.g., in telephone interviews) [10].

To date, the PSS has been translated into more than 20 languages, and its psychometric properties have also been verified in multiple countries. Studies have shown that both the PSS-14 and PSS-10 have satisfactory internal consistency, with alphas ranging from 0.74 [11] to 0.91 [12]; reports of the internal consistency of the PSS-4 have ranged from marginal (0.60) [10] to good (0.82) [12]. The two-factor structure for the PPS-14 and PPS-10 has also been widely validated across cultures [8,10] including China [13,14].

There are two gaps in this literature that are addressed in the current study. First, there have been mixed findings concerning the factor structure of the PSS-4. Most researchers (including the original authors of the PSS) found that a one-factor solution fit the data best [10,15,16]. However, other studies failed to replicate these findings and found that the proposed single-factor of the PSS-4 model indicated a poor fit [17], found that a two-factor solution fit the model best [18], or found that both the one- and two-factor solutions were acceptable [19]. These inconsistent results warrant further investigation. Understanding the factor structure of the PSS-4 will inform future researchers about whether the measure should be regarded as one scale to assess stress level as a whole or can also be used as two subscales similar to the longer forms (PSS-10 and PSS-14).

Second, studies that have compared the reliability, validity, and utility of the three versions of the PSS have been in specific populations, such as college students [16], workers [18], or cardiac patients [20]. The interest of this study was to compare the three versions in a wider population, namely a nationally representative sample in Mainland China, using the Chinese Perceived Stress Scale (CPSS). The 14-item and 10-item versions of the CPSS have good psychometric properties and are widely used in Mainland China [13,14,21,22], but we could not locate any psychometric studies on the CPSS-4. No studies to date have tested whether all three CPSS versions (especially the CPSS-4) are appropriate for use in general samples in Mainland China.

To address these two gaps in the literature, the aim of this study was to assess the construct validity, reliability, and concurrent validity of the three versions of the CPSS, and to compare their appropriateness in a representative sample of adults in Mainland China. Given that the entire world is currently under the burden of COVID-19, more studies are needed to investigate pandemic-related stress and its negative effects. The authors of this study hope to identify a reliable, valid, and feasible version of the CPSS for both research and practical purposes (e.g., for use in telephone interviews and in combination with other measures).

## 2. Materials and Methods

### 2.1. Participants and Procedure

The commercial online survey research firm Wenjuanxing was employed to construct a nationally representative sample from 25 February to 2 March 2020, about one month after the massive outbreak of COVID–19 in China. Wenjuanxing has a large database with information from over 2.6 million Chinese registered users across China. With the permission of customers, Wenjuanxing identified potential participants to create a nationally representative adult community sample based on gender, age, education, and region. The potential participants were contacted by email with information about the study. As an incentive, participants were told that at the end of the study they would receive reward points that could be converted into cash (about USD 0.8 for this survey). In the given period, 1133 response sets were obtained from the online survey platform. This number excluded data from participants who dropped out of the study after completing only part of the survey. Because a forced choice format was used in the survey, there were no missing values in the data collected (completers answered all the items in the survey in the final sample), and data from all 1133 participants were retained for analyses. The final sample included 548 (48.37%) males and 585 (51.63%) females, with ages ranging from 18 to 88 years (*M* = 31.23, *SD* = 8.73). The participants came from 232 cities across 29 provinces and municipalities of Mainland China. Table 1 presents the sociodemographic characteristics of the sample.

Participants were first informed about the research aim, the main content of the questionnaires, and completion time. They were also informed that the questionnaires were anonymous, and research data would only be used for research purposes. If participants consented to participate, they were then directed to the online data collection interface. Participants completed a series of questionnaires, but only the questionnaires related to the current study were analyzed. The study was approved by the Research Ethics Committee of the institution with which the first author is affiliated (HR151-2020).

### 2.2. Measures

#### 2.2.1. Perceived Stress Scale

The full 14-item version of the Perceived Stress Scale includes two subscales: the negativity subscale (items 1, 2, 3, 8, 11, 12, and 14) and the positivity subscale (items 4, 5, 6, 7, 9, 10, and 13) [9]. The 10-item version retained the original two-factor structure: a negativity subscale with six items (items 1, 2, 3, 8, 11, and 14) and a positivity subscale with four items (items 6, 7, 9, and 10) [10]. The PSS-4, the briefest version of the PSS, includes only four items (items 2, 6, 7, and 14). Two items (items 6 and 7) are positively worded and the other two (items 2 and 14) are negatively worded. The original authors of the PSS reported that these four items loaded on a single factor [10].

Participants are asked to rate how often they experienced stressful situations in the past month on a five-point scale, from 0 (never) to 4 (very often). The PSS total score is calculated by reverse-coding the positive items and summing the scores for all items. The total scores range from 0 to 56, from 0 to 40, and from 0 to 16 for the PSS-14, PSS-10, and PSS-4, respectively. A higher score indicates more perceived stress. The PSS-14 was translated to Chinese to create the CPPS-14, which was validated in a sample of cancer survivors [14]. The CPPS-10 and CPPS-4 were created using the same subsets of items used to create the PSS-10 and PSS-4.

#### 2.2.2. Health Questionnaire Depression Nine-Item Scale (PHQ-9)

The PHQ-9 is a nine-item scale designed to measure symptoms of depression [23]. Participants are asked to rate how often they have been bothered by depression-related problems in the past two weeks using a four-point scale, from 0 (not at all) to 3 (nearly every day). The PHQ-9 total score is calculated by adding all nine item scores. The total score ranges from 0 to 27, with a higher score indicating more severe depressive symptoms. The Chinese PHQ-9 has demonstrated good reliability and validity in the general population [24]. In the present study, the internal consistency was Cronbach’s alpha = 0.86.

#### 2.2.3. Generalized Anxiety Disorder-7

The GAD-7 is a seven-item scale designed to measure anxiety symptoms [25]. Participants are asked to assess how often they have been bothered by anxiety-related problems in the past two weeks, using a four-point scale from 0 (not at all) to 3 (nearly every day). The GAD-7 total score is calculated by adding the seven item scores. The total scores range from 0 to 21, with a higher score indicating more severe anxiety symptoms. The GAD-7 has demonstrated good reliability and validity in the Chinese population [26,27]. In the present study, the internal consistency was Cronbach’s alpha = 0.87.

### 2.3. Data Analysis

The data analyses were conducted using the IMB Statistical Package for the Social Sciences software 21.0 (SPSS 21.0) and Mplus version 8.3 [28].

Descriptive statistics, internal consistency reliability, concurrent validity, and group comparisons were calculated in SPSS 21.0. Descriptive statistics were generated as means, standard deviations, and correlations; internal consistency reliability was evaluated by Cronbach’s alpha coefficient; concurrent validity was evaluated based on Pearson’s correlation between the CPSS total score and health-related variables (total scores on the PHQ-9 and GAD-7); group comparisons on gender, age, and educational level were made using t-tests and one-way ANOVAs [18,20]. All participants were split into three age cohorts for purposes of comparison. Given the whole sample was skewed toward the young (*M* = 31.23, *SD* = 8.73), the three age cohorts were labeled younger (less than 26 years old), middle (26–34), and older (35 and above) in this study (a reasonable number was considered in each cohort). All tests of significance were two-tailed, and results were considered significant at *p* < 0.05.

Confirmatory factor analysis (CFA) was conducted in Mplus 8.3 as a test of construct validity. CFAs were performed for each version of the CPSS (CPSS-4, CPSS-10, and CPSS-14). The parameter estimates for the CFAs were obtained using the maximum likelihood method (MLM). For the CFA analysis, the comparative fit index (CFI), the Tucker–Lewis index (TLI), the root mean square error of approximation (RMSEA), and the standardized root mean square residual (SRMR) were used to evaluate model fit. The fit was considered good if RMSEA was below 0.06, SRMR was below 0.08, and CFI and TLI were above 0.95 [29].

## 3. Results

### 3.1. Confirmatory Factor Analysis

This study used CFA to test for the well-established two-factor structure in the two longer versions of the CPSS, CPSS-14 and CPSS-10. In the expected two-factor structure, all positively worded items load on one factor and all negatively worded items load on the other. For the CPPS-4 we used CFA to test both the one-factor model (all four items on one factor) and the two-factor model (two positive items on one factor, and two negative items on the other factor).

Table 2 presents the fit indices for the proposed factor structures of the three versions of the CPSS. All three versions had a two-factor solution. The one-factor solution for the CPPS-4 was not supported in the present sample, as indicated by a poor fit with CFI, TLI, and RMSEA values. However, the two-factor solution for the CPPS-4 showed a near-perfect model fit, with both the CFI and TLI values above 0.95, and the RMSEA and SRMR values close to 0.

The standardized factor loadings of the two-factor models for the CPSS-14, CPSS-10, and CPSS-4 were almost all statistically significant and above 0.50, with the exception of item 12 on the CPSS-14 (with a statistically significant but low factor loading of 0.39 on the CPSS-14). Table 3 presents the standardized factor loadings of the two-factor models for all three versions of the CPPS.

### 3.2. Internal Consistency Reliability

For the CPSS-14, the Cronbach’s alpha was 0.85 for the full scale, 0.82 for the negative subscale, and 0.81 for the positive subscale. The corresponding values for the CPSS-10 were 0.83, 0.83, and 0.72, and for the CPSS-4 they were 0.66, 0.68, and 0.58. All values exceeded 0.60 as the criterion of acceptable internal consistency, except in the case of the positive subscale of the CPSS-4 (0.58).

### 3.3. Concurrent Validity

Concurrent validity was established by calculating the correlations between each of the versions of the CPSS (CPSS-14, CPSS-10, and CPSS-4) and the measures of depression and anxiety (the PHQ-9 and GAD-7, respectively). As presented in Table 4, the total scores on all three versions of the CPSS (CPSS-14, CPSS-10, and CPSS-4) were significantly related to the PHQ-9 and GAD-7 total scores. Concurrent validity was satisfied for all three CPSS versions.

### 3.4. Gender, Age, and Educational Level Differences

Table 5 presents the average total scores on the CPSS-4, CPSS-10, and CPSS-14 by gender, age, and educational level. The results showed that women as a group had a higher average score than men on all three versions of the CPSS (on CPSS-14, *d* = 0.25; on CPSS-10: *d* = 0.25; on CPSS-4, *d* = 0.24). The younger age cohort had a higher average score on perceived stress than the middle age and older age cohorts across the CPSS-14 (younger vs. middle/older cohort, *d* = 0.46), and CPSS-10 (younger vs. middle/older cohort, *d* = 0.39). On the CPSS-4, the average score in the younger cohort was significantly higher than the middle cohort (younger vs. middle cohort, *d* = 0.36) and older cohort (younger vs. older cohort, *d* = 0.43); the average scores for middle and older cohorts were not significantly different from each other (*p* > 0.05, *d* = 0.08). There was no difference in the average scores based on education level on any of the three CPSS measures.

## 4. Discussion

The current study addressed two gaps in the literature on the Chinese Perceived Stress Scale. First, although the CPSS-14 and CPSS-10 have been shown to have good psychometric properties, there have been no psychometric studies on the CPSS-4 in Mainland China. Second, research comparing the psychometric properties of all three versions of the PSS has been conducted in very specific populations, such as cardiac patients [20]. The current study compared the psychometric properties of the three versions of the CPSS in a representative sample in Mainland China.

A two-factor structure of all three versions of the CPSS was well supported by confirmatory factor analysis in this study. These findings echoed those of previous research on the PSS-10 and PSS-14 [8]. However, the two-factor structure for the CPSS-4 was inconsistent with the one-factor structure reported by the original authors of the CPSS-4 [10], and with another study that found both the one-factor and two-factor structures to be acceptable [19]. In fact, the two-factor solution for the CPSS-4 showed a good fit to the data in our sample, and was consistent with a recent study in Italy in which EFA and CFA supported a two-factor structure for the original PSS-4 [18]. The difference in the factor structure across studies may be due to the nature of the samples and the statistical approach [10]. The original PSS-4 had a one-factor solution in college samples [15,16], whereas it had a two-factor solution in community samples [17,18,19,20,30] with the exception of Cohen and Williamson’s original study, which found a one-factor solution in a community sample [10]. However, Cohen and Williamson used EFA to test the PSS-4’s original factor structure [10], and later researchers used CFA to confirm the structure. The present study sampled from a community population and the results supported the two-factor solution for the PSS-4 found in most community samples.

The internal consistencies for the CPSS-14 and CPSS-10 were very good, with Cronbach’s alphas both above 0.80. The Cronbach’s alpha value was lower (0.66) for the CPSS-4, although it was slightly higher than in the original authors’ report (0.60) [10] and was very close to the value reported in research on the CPSS-4 in a Hong Kong sample (0.67) [20]. The internal consistency reliability for the CPSS-4 in the present study is considered acceptable, especially given the small number of items, as the alpha coefficient increases with more items [31]. Given the adequate reliability and well-established two-factor structure for the CPSS-4, the CPSS-4 could be used as two subscales in future research in China.

The concurrent validity of the CPSS was also established in this study. The CPSS-14, CPSS-10, and CPSS-4 all showed strong correlations with the PHQ-9 and GAD-7. The results suggested that more stress perceived by individuals under the COVID-19 pandemic may be linked to higher depression and anxiety symptoms. It is worth noting that the correlation coefficients between the CPSS-4 and the criterion variables (PHQ-9 and GAD-7) were comparable with those of the longer CPSS-10 and CPSS-14, providing further validity information for the four-item CPSS. This should increase researchers’ confidence in using this briefest version in future clinical research and practice.

Furthermore, the present study found gender and age cohort differences on the CPSS. In line with prior work [20], women perceived higher levels of stress than men, while the older population tended to perceive less stress than the younger population. However, the total scores did not vary based on education level, consistent with a study conducted on the original PSS in Italy [18]. It is worth noting that on the CPSS-4 the average scores were highest in the younger cohort (less than 26 years), then trended downward and remained stable in the middle cohort (26–34 years) and the older cohort (more than 34 years). However, this finding must be interpreted with caution because the older cohort had fewer participants and a wider age range than the other cohorts. Future studies may include more participants from the older population to examine the relationship between age cohort and CPSS-4 total score. It will also be important to identify the reasons for the high CPSS scores among the youngest participants. Given that women and the younger population in our sample experienced higher stress under the COVID-19 pandemic, these groups may need more mental health care during this challenging time. In future research the CPSS could be tested as a screening measure for targeted intervention, in line with the idea of personalized medicine proposed in the existing literature [32].

This study has several limitations that should be taken into account when interpreting the results. First, some of the correlations may have been inflated because of shared method variance, as all variables were assessed using self-report questionnaires. Future researchers can use more objective stress measures, such as physiological or behavioral indicators, to investigate the construct validity of the CPSS. Second, this study did not investigate the test–retest reliability of the CPSS. Evidence of this type of reliability would be useful for longitudinal research on the perception of stress. Third, although one of this study’s strengths was the use of a large, nationally representative sample, the subgroup of older adults was relatively small and there were other subgroups that were not examined in the current study, such as health care workers [33]. Finally, the results obtained in this community sample do not provide direct evidence of whether the CPSS will be useful for screening and intervention in a clinical population.

## 5. Conclusions

To our knowledge, this was the first study to examine the factor structure of the CPSS-4 and to compare the psychometric properties of all three versions of the CPSS in a large and representative sample from Mainland China. The CPSS-14, CPSS-10, and CPSS-4 demonstrated adequate to good internal consistency, construct validity, and concurrent validity in the Chinese general population under the high-stress context of the COVID-19 pandemic. In addition, two subscales were identified in all three CPSS versions. All three CPSS versions would be useful for research purposes and may have utility as stress screening measures for mental health intervention in the Chinese population. Given the greater length of the CPSS-14 and the fact that some items on the measure have low factor loadings, and the lower alpha coefficient for the CPSS-4, it appears that the CPSS-10 would be the best choice for general use. However, the CPSS-4 is still an acceptable measure of perceived stress, especially under time constraints (e.g., telephone interviews).

## Figures and Tables

**Table 1:** Sociodemographic characteristics of the sample.

Variables	(*N* = 1133) *n* (%)
Gender	
Males	548 (48.4%)
Females	585 (51.6%)
Age	
≤25	298 (26.3%)
26–34	517 (45.6%)
≥35	318 (28.1%)
Educational level	
Below high school	48 (4.2%)
High school degree	76 (6.7%)
Associate degree	166 (14.7%)
Bachelor degree	740 (65.3%)
Graduate degree	103 (9.1%)

**Table 2:** Goodness of fit indexes for the proposed CPSS models.

	χ^2^	df	*p*	CFI	TLI	RMSEA	SRMR
CPPS-14							
two-factor model	148.266	76	<0.001	0.981	0.977	0.029	0.034
CPPS-10							
two-factor model	65.825	34	<0.001	0.988	0.984	0.029	0.023
CPPS-4							
one-factor model	86.034	2	<0.001	0.865	0.594	0.193	0.064
two-factor model	0.076	1	0.783	1.000	1.009	0.000	0.001

Note: χ^2^, chi-square value; df = degrees of freedom; CFI, comparative fit index; TLI, Tucker–Lewis index; RMSEA, root mean square error of approximation; SRMR, standardized root mean square residual.

**Table 3:** Standardized factor loadings for three versions of the CPSS.

Items	CPSS-14		CPSS-10		CPSS-4	
	Negative	Positive	Negative	Positive	Negative	Positive
1 Upset by something happening unexpectedly?	0.688	0	0.685	0	─	
2 Unable to control the important things in your life?	0.717	0	0.716	0	0.775	0
3 Nervous and stressed?	0.677	0	0.677	0	─	
8 Could not cope with all the things that you had to do?	0.602	0	0.603	0	─	
11 Angered because of things that were outside your control?	0.648	0	0.646	0	─	
12 Thinking about things that you have to accomplish?	0.387	0	─		─	
14 Difficulties were piling up so high that you could not overcome them?	0.710	0	0.709	0	0.662	0
4 Dealt successfully with day-to-day problems and annoyances?	0	0.637		─		─
5 Effectively coping with important changes that were occurring in your life?	0	0.622		─		─
6 Confident about your ability to handle your personal problems?	0	0.687	0	0.649	0	0.710
7 Things were going your way?	0	0.589	0	0.596	0	0.570
9 Dealt successfully with irritating life hassles?	0	0.584	0	0.600		─
10 You were on top of things?	0	0.623	0	0.658		─
13 Able to control the way you spend your time?	0	0.569		─		─
Factor correlation	0.53		0.52		0.56	
Cronbach’s alpha	0.82	0.81	0.83	0.72	0.68	0.58
	0.85		0.83		0.66	

**Table 4:** Correlations between three versions of the CPSS and criterion-related measures.

	CPSS-14	CPSS-10	CPSS-4
PHQ-9	0.61 ***	0.62 ***	0.54 ***
GAD-7	0.59 ***	0.62 ***	0.51 ***

Note: PHQ-9, Patient Health Questionnaire-9; GAD-7, Generalized Anxiety Disorder-7. *** *p* < 0.001.

**Table 5:** Mean differences by gender, age, and educational level.

CPSS-14						CPSS-10					CPSS-4				
	Mean	*SD*	*F/t*	*ES*	*p*	Mean	*SD*	*F/t*	*ES*	*p*	Mean	*SD*	*F/t*	*ES*	*p*
Gender ^a^			4.12	*d* = 0.25	<0.001			4.14	*d* = 0.25	<0.001			4.01	*d* = 0.24	<0.001
Male (*n* = 557)	36.03	7.48				25.72	5.91				9.66	2.68			
Female (*n* = 595)	37.97	8.30				27.24	6.45				10.33	2.91			
Age (years) ^b^ (Mean = 31.23)			2.52	*η*^2^ = 0.09	<0.001			2.10	*η*^2^ = 0.08	<0.001			2.02	*η*^2^ = 0.07	<0.001
≤25 (younger; *n* = 298)	39.62	8.24				28.20	6.40				10.82	2.95			
26–34 (middle; *n* = 517)	36.53	7.70				26.24	6.06				9.80	2.74			
≥35 (older; *n* = 318)	35.41	7.57				25.36	6.06				9.59	2.71			.
Education level ^b^			0.60	*η*^2^ < 0.00	0.67			0.65	*η*^2^ < 0.00	0.63			0.66	*η*^2^ < 0.00	0.63
Below high school (*n* = 48)	38.15	7.54				27.69	6.20				10.65	2.82			
High school (*n* = 76)	37.72	7.79				26.81	6.04				10.03	2.58			
Associate degree (*n* = 166)	37.17	7.34				26.45	5.99				10.00	2.64			
Bachelor (*n* = 740)	36.94	8.02				26.48	6.24				9.98	2.84			
Graduate degree (*n* = 103)	36.35	8.88				26.01	6.80				9.91	3.15			

Note: *SD*, standard deviation; *ES*, effect size. ^a^ The value was calculated with independent *t* tests; ^b^ the value was calculated with one-way analysis of variance (ANOVA).

## Data Availability

The datasets generated during and/or analyzed during the current study are available from the corresponding author on reasonable request.
